# Interpretable Network-Level Biomarker Discovery for Alzheimer’s Stage Assessment Using Resting-State fNIRS Complexity Graphs

**DOI:** 10.3390/brainsci16020239

**Published:** 2026-02-19

**Authors:** Min-Kyoung Kang, Agatha Elisabet, So-Hyeon Yoo, Keum-Shik Hong

**Affiliations:** 1 School of Mechanical Engineering, Pusan National University, Busan 46241, Republic of Korea; mk_kang@pusan.ac.kr (M.-K.K.);; 2 Max Planck Institute for Human Cognitive and Brain Sciences, 04303 Leipzig, Germany; 3 Institute for Future, School of Automation, Qingdao University, Qingdao 266071, China

**Keywords:** Alzheimer’s disease, functional near-infrared spectroscopy (fNIRS), graph neural networks, signal complexity, resting-state brain networks

## Abstract

**Highlights:**

**What are the main findings?**

- Complexity–fluctuation graphs outperform amplitude-based functional connectivity in resting fNIRS.
- Reproducible prefrontal network alterations are most prominent at the MCI stage.

**What are the implications of the main findings?**

- Graph-based resting-state fNIRS enables interpretable, task-free Alzheimer’s assessment.
- MCI-specific network disruptions highlight targets for early detection and monitoring.

**Abstract:**

**Background/Objectives**: This study introduces a reproducible and interpretable graph-based framework for resting-state functional near-infrared spectroscopy (fNIRS) that enables network-level biomarker discovery for Alzheimer’s disease (AD). Although resting-state fNIRS is well suited for task-free assessment, most existing approaches rely on static channel-wise features or conventional functional connectivity, limiting insight into coordinated network dynamics and reproducibility. **Methods**: Resting-state prefrontal fNIRS signals were represented as subject-level graphs in which edges captured coordinated fluctuations of nonlinear signal complexity across channels, computed using sliding-window analysis. Graph neural networks (GNNs) were employed as analytical tools to identify disease-stage-related network patterns. Interpretability was assessed using edge-level importance measures, and reproducibility was evaluated through fold-wise stability analysis and consensus network construction. **Results**: The proposed complexity–fluctuation-based graph representation consistently outperformed conventional amplitude-based functional connectivity. Statistically supported prefrontal network biomarkers distinguishing mild cognitive impairment (MCI) from healthy aging were identified, with statistically significant group differences (*p* = 0.001). In contrast, network patterns associated with Alzheimer’s disease were more heterogeneous and less consistently expressed. Consensus analysis revealed a subset of prefrontal connections repeatedly selected across cross-validation folds, and attention-based network patterns showed strong spatial correspondence with statistically derived biomarkers. **Conclusions**: This study establishes a reproducible and interpretable framework for resting-state fNIRS analysis that emphasizes coordinated complexity dynamics rather than classification accuracy. The results indicate that network-level alterations are most consistently expressed at the MCI stage, highlighting its role as a critical transitional state and supporting the potential of the proposed approach for longitudinal monitoring and clinically applicable fNIRS-based assessment of neurodegenerative disease.

## 1. Introduction

Alzheimer’s disease (AD) is a progressive neurodegenerative disorder characterized by a gradual decline in cognitive function, typically evolving from normal aging through mild cognitive impairment (MCI) to dementia [1,2,3]. Because pathological changes such as amyloid-β deposition, tau pathology, synaptic loss, and neurovascular dysfunction precede overt clinical symptoms by many years, early-stage assessment and longitudinal monitoring across the Alzheimer’s continuum are essential for timely intervention and disease management [4,5]. In this context, neuroimaging biomarkers that are non-invasive, repeatable, and sensitive to subtle functional alterations are of increasing importance [6,7].

Functional near-infrared spectroscopy (fNIRS) has emerged as a promising modality for studying neurovascular dynamics in aging and dementia [8,9]. Compared to magnetic resonance imaging (MRI) or positron emission tomography (PET), fNIRS offers practical advantages including low cost, portability, and tolerance for repeated measurements, making it particularly suitable for longitudinal and real-world monitoring [6,9]. Notably, fNIRS is well suited for probing prefrontal cortex (PFC) function, a region known to be vulnerable in early AD and closely associated with executive function, working memory, and attentional control [10,11].

Despite these advantages, most existing fNIRS studies in AD rely on task-evoked paradigms and channel-wise static features [12,13]. Cognitive tasks such as n-back, Stroop, or verbal fluency are often used to elicit prefrontal activation differences between healthy controls and cognitively impaired individuals [13,14]. However, task-based approaches impose a substantial cognitive burden on MCI and AD patients, suffer from large inter-subject variability in task performance, and limit feasibility for frequent or long-term monitoring [9,15]. Moreover, static channel-wise metrics—such as mean or peak hemodynamic responses—provide limited insight into network-level reorganization and may be confounded by systemic physiological noise [16,17].

Resting-state paradigms provide an attractive alternative by eliminating task demands and enabling standardized, repeatable measurements across patient populations [18,19]. In resting-state fNIRS, functional connectivity is typically estimated via Pearson correlation of raw hemodynamic time series, yielding amplitude-based functional connectivity (FC) networks [19,20]. While such approaches have demonstrated group-level differences in AD and MCI, they predominantly capture linear amplitude coupling and may fail to reflect alterations in the intrinsic dynamical regulation of neurovascular signals that accompany neurodegeneration [17,21]. Furthermore, resting-state fNIRS signals are inherently characterized by a lower signal-to-noise ratio (SNR) compared to task-evoked paradigms and are susceptible to diverse systemic physiological noise. These factors, combined with the significant biological heterogeneity across the Alzheimer’s continuum, often lead to increased variability in model performance and hinder the establishment of stable neuroimaging biomarkers. Despite these challenges, developing robust task-free assessment frameworks remains a clinical priority to minimize the cognitive burden on elderly patients and enable longitudinal monitoring.

Accumulating evidence from nonlinear dynamics and complexity science suggests that neurodegenerative diseases are associated not only with changes in signal amplitude but also with altered temporal structure, irregularity, and adaptability of brain signals [22,23]. Measures such as spectral entropy, fractal dimension, and wavelet entropy have been shown to capture disease-related changes in neural complexity and have been increasingly applied to fNIRS data [24,25,26]. However, prior studies largely analyze these complexity measures at the channel level, without explicitly modeling how complexity fluctuations are coordinated across distributed cortical regions [17,27].

From a network neuroscience perspective, Alzheimer’s disease is increasingly understood as a disorder of disrupted large-scale interactions rather than isolated regional dysfunction [28,29]. Consequently, there is a critical need for network-aware representations that can integrate nonlinear signal characteristics with inter-regional coupling, particularly in task-free resting-state conditions [16,27]. Graph-based modeling provides a natural framework for this purpose, where brain regions are represented as nodes and their interactions as edges [30]. Recent advances in graph neural networks (GNNs) have further enabled data-driven learning on such graph-structured representations, offering the ability to jointly model node attributes and network topology [31,32].

However, applying GNNs to clinical neuroimaging data raises several important challenges [33]. First, improvements in classification accuracy alone are insufficient for translational relevance; interpretability is essential to identify candidate biomarkers that align with known neurophysiological mechanisms [33,34]. Second, many explainable AI approaches yield visually appealing importance maps but do not assess the reproducibility or stability of identified biomarkers across data splits, limiting their scientific reliability [35,36]. Finally, it remains unclear how alternative graph construction strategies—particularly those based on nonlinear signal dynamics rather than raw amplitude correlations—affect both model behavior and interpretability in resting-state fNIRS [17,26]. This concern echoes recent “trustworthy” neurodegenerative-disease detection studies (e.g., ChiGa-Net), where refined feature selection (χ^2^ scoring) and robust optimization (genetic search) are used to strengthen reliability and generalization [37].

**Figure 1:**
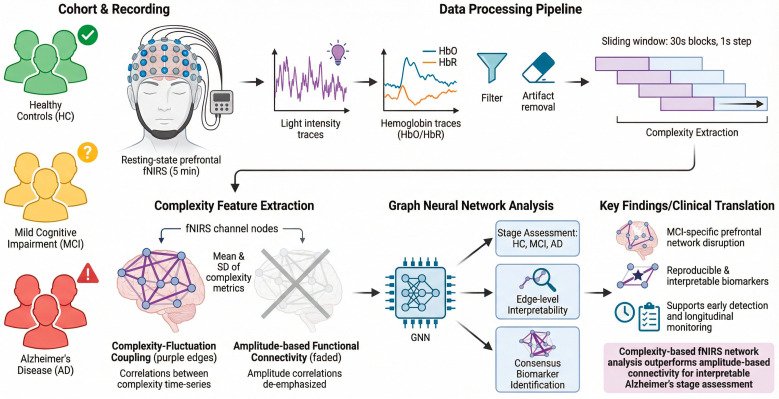
fNIRS-based graph neural network framework for Alzheimer’s disease biomarker discovery.

In this study, we introduce an interpretable, network-based framework for Alzheimer’s stage assessment using resting-state prefrontal fNIRS. Subject-level graphs are constructed in which nodes correspond to fNIRS channels and edges represent complexity–fluctuation coupling, defined as inter-channel correlations of sliding-window-derived nonlinear complexity metrics. Unlike conventional functional connectivity based on raw HbO/HbR amplitudes, this approach models coordinated modulation of signal dynamics, capturing inter-regional synchronization of temporal complexity patterns. Graph neural networks are then applied to learn stage-related network patterns across healthy controls, MCI, and AD, while explainability analyses identify influential connections (Figure 1).

Crucially, we go beyond model performance to emphasize reproducible interpretability. By systematically comparing raw functional connectivity graphs with complexity-derived graphs, performing feature ablation analyses, and evaluating fold-wise stability and consensus of important edges, we derive network-level biomarker candidates that are robust across cross-validation folds. This approach aligns with the growing demand for explainable and reliable AI methods in neuroscience and supports the development of task-free, low-burden biomarkers suitable for longitudinal monitoring and future neuroadaptive systems.

In summary, this study introduces a dynamics-informed graph-based framework for resting-state fNIRS analysis and demonstrates its utility for reproducible, network-level biomarker discovery in Alzheimer’s disease.

By modeling coordinated complexity fluctuations, leveraging interpretable graph neural networks, and explicitly evaluating fold-wise stability, the proposed approach identifies robust prefrontal network alterations that are most consistently expressed at the mild cognitive impairment stage.

## 2. Method

### 2.1. Participants

The dataset comprised resting-state functional near-infrared spectroscopy (fNIRS) recordings from three groups: patients with Alzheimer’s disease (AD), patients with mild cognitive impairment (MCI), and cognitively healthy controls (HC). All participants were recruited through Pusan National University Hospital, Republic of Korea, following established clinical diagnostic criteria. AD and MCI diagnoses were confirmed by experienced clinicians using comprehensive neuropsychological assessments, including the Korean version of the Mini-Mental State Examination (K-MMSE), Korean-Montreal Cognitive Assessment (K-MoCA), Clinical Dementia Rating (CDR), and supporting neuroimaging when available. Healthy controls were recruited from the local community and had no history of neurological or psychiatric disorders. All participants were right-handed, native Korean speakers, and provided written informed consent prior to participation. The study protocol was approved by the Institutional Review Board of Pusan National University Hospital and conducted in accordance with the Declaration of Helsinki. Demographic characteristics, including age, sex, education level, and cognitive scores, are summarized in Table 1. Detailed recruitment procedures and diagnostic criteria are consistent with those reported previously.

To maintain methodological consistency with our previous study utilizing the same cohort [38], clinical and demographic comparisons were strictly controlled for potential age-related confounding effects. Although a significant age difference was observed between groups (*p* < 0.01), we implemented a rigorous statistical control protocol to ensure that cognitive deficits were independent of chronological aging. Specifically, Analysis of Covariance (ANCOVA) was performed using the MATLAB R2024a (The MathWorks, Inc., Natick, MA, USA) function aoctool.m, with age included as a nuisance covariate. This analysis confirmed that the effect of the diagnostic group (AD vs. MCI vs. HC) on cognitive performance remained statistically significant after adjusting for age, validating the disease-specific nature of the observed impairments.

### 2.2. fNIRS System and Data Acquisition

**Figure 2:**
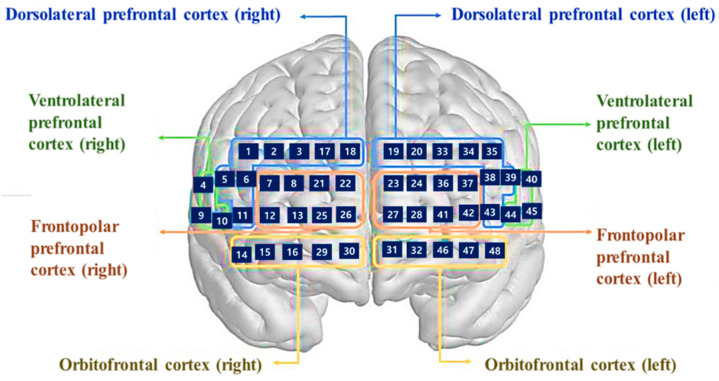
48 channel optodes configuration of the NIRSIT device [39]. (OBELAB, NIRSIT Analysis Tool Manual, 2021).

Resting-state fNIRS data were acquired using a continuous-wave, wearable fNIRS system (NIRSIT; OBELAB Inc., Seoul, Republic of Korea). The system consists of 24 light sources and 32 detectors arranged over the prefrontal cortex, yielding a dense montage with multiple source–detector pairs. Near-infrared light at wavelengths of 780 nm and 850 nm was used, and signals were sampled at 8.138 Hz. The device was positioned on the participant’s forehead relative to the Fpz location of the international 10–20 system, covering bilateral dorsolateral, ventrolateral, frontopolar, and orbitofrontal regions, see Figure 2.

From the full montage, 48 channels were selected for analysis to focus on cortical hemodynamics and minimize superficial contamination. Anatomical correspondence of each channel was determined a priori based on the device layout and standard prefrontal subdivisions. Participants were seated comfortably in a quiet, dimly lit room and instructed to remain still, relaxed, and awake during a resting-state recording lasting approximately 5 min. No explicit cognitive task was performed. Acquisition conditions and hardware settings followed the same protocol as previously described.

### 2.3. Preprocessing

Raw light intensity signals were visually inspected to identify channels with poor signal quality. Motion artifacts were detected using a sliding-window-based motion index, and segments exceeding predefined thresholds were masked. Channels were excluded if motion-contaminated segments exceeded 10% of the total recording duration. Intensity signals were converted into concentration changes of oxygenated hemoglobin (HbO) and deoxygenated hemoglobin (HbR) using the modified Beer–Lambert law. To isolate physiologically relevant spontaneous fluctuations, a fourth-order Butterworth band-pass filter (0.0018–0.15 Hz) was applied to all hemoglobin signals. This frequency range preserves endothelial, neurogenic, and myogenic oscillations while attenuating very slow drift and high-frequency cardiac and respiratory components. All preprocessing steps followed established procedures reported in prior work using the same dataset and device.

### 2.4. Complexity Metrics

To characterize intrinsic hemodynamic dynamics, three nonlinear complexity measures were extracted from each preprocessed channel: Higuchi’s fractal dimension (HFD), spectral entropy (SE), and wavelet entropy (WE). These metrics capture complementary aspects of temporal irregularity, spectral unpredictability, and multiscale organization.

Unlike conventional static feature extraction, complexity measures were computed in a sliding-window manner to capture temporal fluctuations. Each 250 s resting-state time series was segmented into overlapping windows of 30 s with a 1 s step. The 30 s window length was selected to strike a balance between capturing stable local signal statistics for non-linear complexity measures and maintaining sufficient temporal resolution to track fluctuations in brain states, as suggested in previous resting-state fNIRS studies. This 1 s step size was chosen to ensure high temporal overlap, thereby preserving fine-grained fluctuations in complexity trajectories and minimizing the loss of transient information that might otherwise be smeared by fixed-window analysis. Complexity metrics were then computed independently within each window for each channel and hemoglobin type (HbO, HbR), yielding time series of complexity values that reflect window-to-window variability in signal dynamics.

HFD quantifies the self-similarity and complexity of a time series. For a windowed signal *x*^(*w*)^, the length *L*(*k*) at scale *k* is defined as

(1) $$L_{m}(k)=\frac{1}{k}\cdot \frac{\left(N-1\right)}{[\frac{N-m}{k}]k}\sum_{i=1}^{\left[\frac{N-m}{k}\right]}\left|x(m+ik)-x(m+(i-1)k)\right|.$$

The fractal dimension is estimated as the slope of the linear regression:

(2) $$HFD=\frac{d\log L(k)}{d\log(1/k)}.$$

SE measures the unpredictability of signal power across frequencies. Let *P*(*f*) denote the power spectral density estimated via Welch’s method. The normalized spectrum is

(3) $$P^{\prime }(f)=\frac{P(f)}{\sum_{f}P(f)}.$$

SE is then computed as

(4) $$SE=-\sum_{f=1}^{n}P^{\prime }(f)\cdot \log P^{\prime }(f),$$

and normalized by log_2_(*F*), where *F* is the number of frequency bins. WE captures multiscale signal irregularity. Given discrete wavelet coefficients {*c_j_*} at scale *j*, the energy at each scale is

(5) $$E_{j}=\sum_{i}\;{\left|c_{i,j}\right|}^{2}.$$

(6) $$p_{j}=\frac{E_{j}}{\sum E_{j}}.$$

Wavelet entropy is defined as

(7) $$WE=-\sum_{j}p_{i,j}\log p_{i,j},$$

and normalized by log_2_(*J*), where *J* is the number of decomposition levels. Parameter settings for each metric were selected based on established recommendations and were identical across subjects to ensure comparability.

To isolate the effect of disease progression from potential age-related confounding, an ANCOVA was performed on the extracted complexity features. In this model, the disease stage (HC, MCI, AD) was treated as the main independent variable, while chronological age was included as a covariate. This approach allows for the identification of hemodynamic complexity alterations that are uniquely attributable to the pathological state rather than normal biological aging. For all ANCOVA models, the *F*-statistic and corresponding *p*-values for the group effect were calculated for each fNIRS channel. The False Discovery Rate (FDR) was controlled using the Benjamini–Hochberg procedure, with statistical significance defined at *q* < 0.05. As a validation step, we confirmed that no statistically significant linear correlation existed between age and the selected complexity metrics (*r* < 0.3, *p* > 0.05) before proceeding with the age-adjusted graph analysis.

### 2.5. Complexity–Fluctuation Coupling Graphs

For each subject, a subject-level graph was constructed to represent network interactions among prefrontal channels based on complexity dynamics. Each node corresponded to one fNIRS channel, and node attributes consisted of summary statistics (mean and standard deviation) of the windowed complexity metrics.

Edges were defined as Pearson correlations between sliding-window complexity time series (HFD, SE, WE) from pairs of channels, forming weighted undirected adjacency matrices. Specifically, for each pair of channels, the Pearson correlation coefficient was computed between their windowed complexity time series. Although Pearson correlation is a linear measure, its use in this study does not imply an assumption of linear neural dynamics. Nonlinear characteristics of resting-state brain activity are explicitly captured at the node level through complexity metrics (HFD, SE, and WE). The role of the edges is instead to quantify inter-regional coupling between the temporal fluctuations of these nonlinear dynamical states.

By applying Pearson correlation to windowed complexity trajectories rather than raw hemodynamic signals, the proposed edge definition measures the degree of co-fluctuation (co-modulation) of complexity states across channels over time. This approach provides a robust and interpretable estimate of dynamic coordination between regions, while avoiding over-parameterization and instability that may arise from applying nonlinear coupling measures to short sliding-window sequences. Accordingly, the resulting graphs emphasize coordination in dynamical state transitions rather than amplitude-based coupling.

To ensure a comprehensive evaluation of neurovascular dynamics, separate graphs were systematically constructed and evaluated using oxygenated hemoglobin (HbO), deoxygenated hemoglobin (HbR), and a combination of both (Both HbO and HbR). This data-driven approach allowed for the comparative assessment of different chromophores and their respective contributions to disease-stage discrimination. Furthermore, graphs were constructed for different edge definitions: (i) traditional amplitude-based functional connectivity derived from raw hemoglobin time series (RAW_FC), and (ii) complexity–fluctuation coupling graphs derived from HFD, SE, and WE time series (COMPLEXITY_FC).

To ensure the reliability of the network representation, correlation matrices were thresholded using a fixed absolute value of |*r*| = 0.3 before being converted into weighted, undirected graphs. A systematic threshold sensitivity analysis was conducted to verify that the observed network topology was not artificially driven by an arbitrary binarization threshold (Supplementary Figure S1). The absolute correlation threshold |*r*| was varied from 0.2 to 0.6 in increments of 0.05. For each threshold, adjacency matrices were reconstructed and the following graph-theoretical metrics were computed: network density, mean degree, clustering coefficient, global efficiency, isolated node ratio, and giant component ratio (GCC). Based on these observations, |*r*| = 0.3 was selected as it lies within a stable topological regime, effectively filtering weak correlations while preserving coherent network organization.

### 2.6. Graph Neural Network Architecture and Training

GNNs were employed to learn stage-related patterns from the constructed subject-level graphs. Each graph was represented by its adjacency matrix (edge weights) and node feature matrix. We evaluated multiple GNN architectures, including graph convolutional networks (GCN) and graph attention networks (GATs), to account for both topological structure and node-level attributes. The detailed architectural configurations and hyperparameter settings of the GCN, GAT, and STGNN models are summarized in Table 2. Models were trained to perform multiclass classification across AD, MCI, and HC groups. Training employed stratified subject independent 5-fold cross-validation to ensure balanced representation of diagnostic groups in each fold. All feature normalization and scaling were performed within training folds only to prevent data leakage. Class imbalance was addressed using class-weighted loss functions.

### 2.7. Interpretable Biomarker Identification and Stability Analysis

To move beyond classification performance and identify biologically meaningful network biomarkers, we employed an integrated framework combining model explainability, stability analysis, and statistical validation. This framework was designed to extract reproducible, stage-related network connections from GNN models trained on resting-state fNIRS graphs.

#### 2.7.1. Edge-Level Explainability

For a trained GNN model, edge-level importance scores were estimated to quantify the contribution of each connection to the model’s prediction. Let *G* = (*V*, *E*) denote a subject-level graph, where *V* represents fNIRS channels and *E* represents inter-channel connections. For each edge *e_ij_* ∈ *E*, an importance score *ϕ_ij_* was obtained using model-specific explainability techniques.

For graph attention networks (GATs), attention coefficients *α_ij_* learned during message passing were served as preliminary indicators for potential biomarkers

(8) $$\phi_{ij}^{GAT}=\frac{1}{H}\sum_{h=1}^{H}\alpha_{ij}^{\left(h\right)},$$

where *H* denotes the number of attention heads.

For non-attention-based models, post hoc explainer algorithms were applied to estimate the contribution of each edge to the model output. These approaches approximate the influence of an edge by optimizing a soft edge mask that maximizes the mutual information between the original model prediction and the masked graph. Edges with the highest importance scores were extracted for each diagnostic group and visualized as class-specific subgraphs.

To avoid interpreting attention scores as causal importance, we further validated edge importance using a perturbation-based sensitivity test. Specifically, for each trained model we ranked edges by the obtained importance scores (attention coefficients or explainer-derived scores for non-attention models) and removed the top-K (=10%) fraction of edges from the input graph (edge deletion). We quantified the resulting change in classification performance (Δ metric) and compared it against a null distribution generated by removing the same number of randomly selected edges repeated N = 1000 times. An empirical *p*-value was computed as the proportion of random trials yielding performance degradation equal to or larger than the top-K deletion.

#### 2.7.2. Fold-Wise Stability and Consensus Network Construction

To assess the robustness of identified biomarkers, edge importance analysis was performed independently for each cross-validation fold. For fold *k*, let *E_k_* denote the set of top-ranked edges. Stability between folds *k* and *l* was quantified using the Jaccard similarity coefficient:

(9) $$Jaccard(E_{k},E_{l})=\frac{\left|E_{k}\cap E_{l}\right|}{\left|E_{k}\cup E_{l}\right|},$$

Edges that appeared consistently across folds were considered more reliable candidates. A consensus network was constructed by aggregating edges that occurred in at least a proportion *ρ* of folds as follows.

(10) $$E_{consensus}=\{e|\frac{1}{K}\sum^{K}I(e\in E_{k})\geq \rho \},$$

where *K* is the number of folds and *I*(⋅) is the indicator function. This consensus representation highlights network connections that are robust to data partitioning and model variability.

Finally, to ensure that the identified consensus network was not a product of random chance, a non-parametric permutation test with 5000 iterations was conducted [27]. For each iteration, the diagnostic labels (HC, MCI, and AD) were randomly shuffled, and the entire pipeline, including GNN training, fold-wise importance extraction, and consensus construction, was repeated to generate a null distribution of fold-support frequencies. The *p*-value (*p_perm_*) for each edge was derived as the proportion of permutations yielding support equal to or greater than the observed values. Multiple comparisons across the network were controlled using the Benjamini–Hochberg FDR procedure, with a significance threshold of *q* < 0.05.

#### 2.7.3. Statistical Validation of Candidate Network Biomarkers

To further validate the identified candidate biomarkers, statistical analyses were conducted on edge importance distributions across diagnostic groups. For each candidate edge *e_ij_*, importance scores {*ϕ_ij_*^(*g*)^} were collected for each group *g* ∈ {HC, MCI, AD}.

Pairwise group comparisons were performed using Welch’s *t*-test when normality assumptions were satisfied, and nonparametric alternatives otherwise. For three-group comparisons, the Kruskal–Wallis test was applied as follows.

(11) $$H=\frac{12}{N(N+1)}\sum_{g}n_{g}({\bar{R}}_{g}-\bar{R}{)}^{2},$$

where *n_g_* and ${\bar{R}}_{g}$ denote the sample size and mean rank of group *g*, respectively. Statistical significance was defined at *p* < 0.05, with correction for multiple comparisons when appropriate.

## 3. Results

### 3.1. Effects of Graph Representation Design

The dataset consisted of resting-state prefrontal fNIRS recordings from HC, MCI, and AD. Group-wise dataset characteristics, including the number of subjects, recording length, and the number of extracted sliding windows, are summarized in Table 3.

#### 3.1.1. Performance Comparison Between RAW_FC and COMPLEXITY_FC

To examine how graph representation choices influenced downstream disease-stage decoding, combinations of node feature compositions and edge construction strategies were systematically evaluated. Node features were constructed using eight predefined feature sets: FS0_MS (mean, standard deviation), FS1_MS_SE, FS2_MS_HFD, FS3_MS_WE, FS4_MS_SE_HFD, FS5_MS_SE_WE, FS6_MS_HFD_WE, and FS7_MS_SE_HFD_WE, which progressively incorporated nonlinear complexity metrics alongside basic statistical descriptors. Graph edges were constructed either from raw time-series correlations (RAW_FC) or from correlations of window-wise complexity fluctuations (COMPLEXITY_FC). The detailed results are provided in Supplementary Table S1.

**Figure 3:**
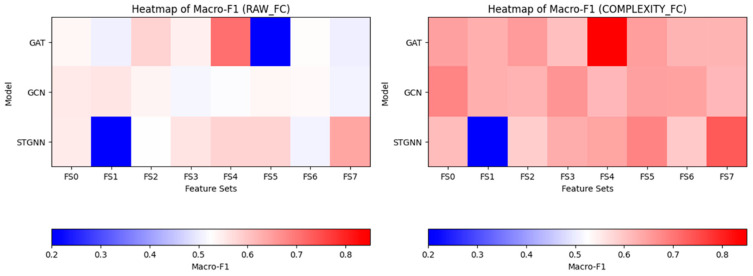
Heatmap of Macro-F1 across feature sets and edge types.

Across all models and feature configurations, complexity-based edge construction consistently outperformed raw connectivity (Figure 3). For example, the GAT model achieved a Macro-F1 score of 0.846 ± 0.060 using FS4 under COMPLEXITY_FC, compared to 0.710 ± 0.040 under RAW_FC with the same feature set. Similar performance gains were observed for GCN (e.g., 0.682 ± 0.039 vs. 0.555 ± 0.024 for FS0) and STGNN (e.g., 0.735 ± 0.090 vs. 0.640 ± 0.040 for FS7), demonstrating the general benefit of complexity-informed graph topology.

Feature composition also exerted a substantial influence on performance. Feature sets integrating both spectral entropy and Higuchi fractal dimension (FS4) consistently yielded the highest Macro-F1 scores and robust MCI recall across models, whereas baseline statistical features alone (FS0_MS) showed inferior performance. In contrast, certain configurations exhibited extreme class-specific sensitivity, such as FS5 under RAW_FC in the GAT model, which achieved perfect MCI recall (1.00) but markedly reduced overall Macro-F1 (0.200 ± 0.020), reflecting severe class imbalance effects.

Together, these results demonstrate that both node-level feature composition and edge-definition strategy are critical determinants of graph representation quality and substantially shape downstream disease-stage discrimination.

#### 3.1.2. Topological Differences Between RAW_FC and COMPLEXITY_FC Graphs

**Figure 4:**
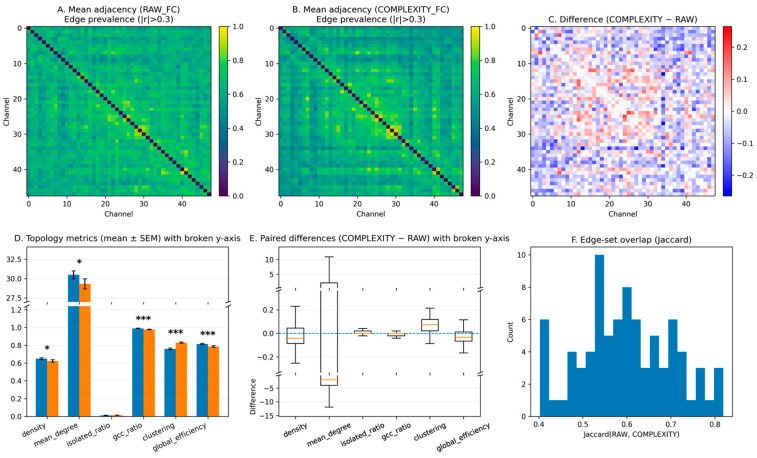
(**A**) Mean adjacency matrix of RAW_FC (edge prevalence |*r*| > 0.3). (**B**) Mean adjacency matrix of COMPLEXITY_FC. (**C**) Difference map (COMPLEXITY – RAW), where red indicates higher connectivity in COMPLEXITY_FC and blue indicates higher connectivity in RAW_FC. (**D**) Topological metrics (mean ± SEM) with broken y-axis. Blue bars represent RAW_FC and orange bars represent COMPLEXITY_FC. (**E**) Paired subject-level differences (COMPLEXITY–RAW). (**F**) Distribution of edge-set overlap measured using the Jaccard similarity coefficient. For adjacency matrices (**A**,**B**), color intensity represents edge prevalence (0–1). In the difference map (**C**), warm colors indicate positive differences and cool colors indicate negative differences. Asterisks denote statistical significance (* *p* < 0.05; *** *p* < 0.001).

To investigate whether the performance advantage of complexity-based graph representations is accompanied by systematic structural differences, we compared subject-level network topology between RAW_FC and COMPLEXITY_FC graphs using an identical absolute threshold (|*r*| = 0.3). The results are summarized in Figure 4, which integrates mean adjacency patterns, paired topology metrics, and edge-set overlap.

Visually, the mean adjacency matrix of RAW_FC (Figure 4A) exhibited broadly elevated connectivity distributed across most channel pairs, resulting in a relatively diffuse appearance. In contrast, the COMPLEXITY_FC adjacency matrix (Figure 4B) showed more sharply delineated block-like patterns, suggesting locally concentrated clusters of coordinated connections rather than uniformly distributed coupling. The difference map (COMPLEXITY–RAW) indicated non-uniform, region-specific rewiring across prefrontal channels. At the subject level, edge-set overlap analysis showed a moderate Jaccard similarity distribution (approximately 0.4–0.8; Figure 4F), suggesting partial preservation of conventional amplitude-based functional connectivity alongside the introduction of distinct complexity-driven connections. Paired statistical comparisons of graph topology metrics (Figure 4D,E) demonstrated that COMPLEXITY_FC graphs were significantly sparser than RAW_FC graphs, with reduced network density (*p* = 0.0153) and mean degree (*p* = 0.0157). Despite this reduction, the isolated-node ratio did not differ significantly between graph types (*p* = 0.27), indicating that the thresholding strategy did not introduce excessive network fragmentation.

Importantly, COMPLEXITY_FC graphs exhibited a significantly lower giant component ratio (*p* = 1.04 × 10^−6^) and reduced global efficiency (*p* = 2.11 × 10^−5^), reflecting diminished global integration. In contrast, the global clustering coefficient was significantly higher in COMPLEXITY_FC than in RAW_FC (*p* = 2.20 × 10^−11^), indicating enhanced local segregation. These findings show that complexity–fluctuation coupling yields a distinct network topology characterized by increased local clustering and reduced global integration, rather than a simple rescaling of amplitude-based functional connectivity. This structural reorganization provides a mechanistic basis for the improved disease-stage discrimination achieved by complexity-based graph representations.

### 3.2. Model-Dependent Performance Characteristics and Selection

To guide model selection for subsequent biomarker analysis, we examined representative configurations exhibiting high sensitivity to mild cognitive impairment (MCI) across different graph neural network architectures, edge construction strategies, and hemoglobin signal types (HbO, HbR, and Both) (Table 4). The performance was systematically evaluated to identify how different chromophores influence the model’s ability to distinguish between disease stages.

Several configurations achieved near-perfect MCI recall (≈1.00), most notably the STGNN model using complexity-based connectivity with both HbO and HbR signals (FS1) and the GAT model using raw connectivity with HbO signals (FS5). However, these configurations were accompanied by markedly reduced Macro-F1 scores (≈0.20), indicating a collapse toward single-class prediction and poor discrimination of healthy control and Alzheimer’s disease groups.

In contrast, the GAT model with complexity-based functional connectivity using HbO signals and the FS4 feature set demonstrated a favorable balance between MCI sensitivity and overall classification performance. This configuration achieved an MCI recall ranging from 0.75 to 0.85, together with the highest observed Macro-F1 score (≈0.846), without evidence of class collapse. The GCN model under the same configuration using both HbO and HbR signals (Both) showed more conservative but stable performance, whereas STGNN with both signals (FS7) exhibited balanced sensitivity with higher variability across folds. To ensure transparency regarding the stability of our findings, we provided the confusion matrices for each cross-validation fold in the Supplementary Figure S2. As shown in Figure S2, the diagonal elements remained dominant across all folds, confirming that the high Macro-F1 score (0.846) was not driven by a single high-performing fold but represented a consistent classification trend across the dataset.

Based on this comparative analysis, the GAT model with HbO complexity-based connectivity and FS4 was selected for subsequent interpretable biomarker extraction, as it provided a robust trade-off between sensitivity to the intermediate disease stage and stable multi-class discrimination.

### 3.3. Interpretable Graph Biomarkers and Network-Level Patterns

**Figure 5:**
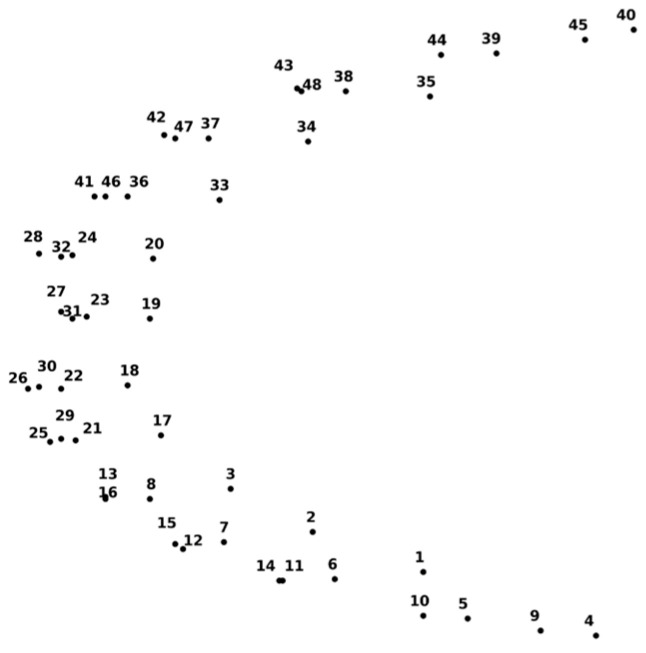
Graph channel configuration.

This section investigates the interpretable network-level biomarkers learned by the proposed graph neural network framework under the selected configuration (GAT with complexity-based functional connectivity using HbO signals and FS4 feature set). Specifically, we analyze (i) pairwise group-discriminative graph biomarkers, (ii) fold-wise stability and consensus networks, and (iii) attention-difference patterns to characterize disease-stage-dependent prefrontal network reorganization. Figure 5 shows the graph channel configuration.

#### 3.3.1. Pairwise Graph Biomarkers for Disease-Stage Differentiation

**Figure 6:**
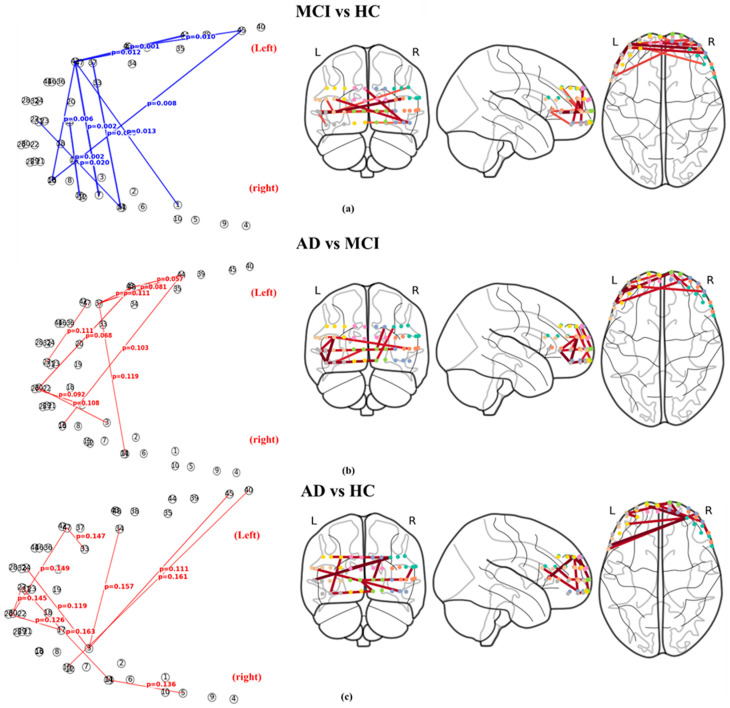
Top-10 graph biomarkers for each pairwise group comparison. (**a**) MCI vs. HC: Blue edges indicate connections with higher edge-importance values in HC. (**b**) AD vs. MCI: Red edges indicate connections with higher edge-importance values in AD. (**c**) AD vs. HC: Red edges indicate connections with higher edge-importance values in AD. For each comparison, the left panel shows the network graph representation, and the right panels illustrate the spatial distribution of significant connections on 3D brain templates, displayed in frontal, lateral, and inferior views. Nodes represent fNIRS channels, and edge labels denote Welch’s *t*-test *p*-values.

- MCI vs. HC: In the comparison between MCI and healthy controls (HC), the top-ranked graph connections consistently exhibited lower edge-importance values in the MCI group (Diff = Mean*_MCI_* − Mean*_HC_* < 0). Among the top 10 discriminative edges, all connections showed negative differences, indicating reduced network-level coordination in MCI. The smallest observed *p*-value was *p* = 0.001, with *p*-values across the top 10 edges ranging from 0.001 to 0.014 (Welch’s *t*-test). These connections primarily involved frontopolar–ventrolateral prefrontal channels, 42–44; dorsolateral–ventrolateral prefrontal channels, including 39–44 and 38–40, which were repeatedly identified as discriminative features. Figure 6a visualizes these results, where blue edges denote connections with higher importance in HC. The spatial distribution indicates a systematic attenuation of complexity-based inter-channel coupling in the MCI group.
- AD vs. MCI: In the AD vs. MCI comparison, most top-ranked connections exhibited higher edge-importance values in AD (Diff = Mean*_AD_* − Mean*_MCI_* > 0). However, statistical separation was weaker than that observed in the MCI–HC comparison. The minimum *p*-value was *p* = 0.054, and all top-ranked edges showed *p*-values above the conventional significance threshold. This pattern suggests a trend toward reorganization or re-amplification of network-level complexity interactions in AD, albeit with substantial inter-subject variability. Figure 6b presents the spatial distribution of these connections, with red edges indicating higher importance in AD.
- AD vs. HC: Similarly, the AD vs. HC comparison showed higher edge-importance values in AD for most top-ranked connections. However, all *p*-values exceeded 0.11, indicating limited statistical separability between these groups based on the current resting-state complexity graphs. Figure 6c illustrates these results, highlighting that while AD-related increases in network importance are present, they do not form strongly discriminative patterns at the group level.

To identify connections exhibiting group-level differences across all three diagnostic categories, a Kruskal–Wallis test was applied. Among the screened connections, the smallest *p*-value was observed for the 13–44 connection (H = 9.91, *p* = 0.007). The mean edge-importance values for this connection followed a non-monotonic pattern, with lower values in MCI relative to HC and increased values in AD. This result indicates that certain prefrontal connections exhibit stage-dependent modulation rather than a simple linear progression across disease stages.

#### 3.3.2. Fold-Wise Stability and Consensus Network

The stability of graph biomarkers across cross-validation folds was assessed using Jaccard similarity between fold-specific sets of important edges. The Jaccard index showed a low but non-zero overlap (mean = 0.104, SD = 0.025), indicating that only a subset of edges was consistently selected across folds. To verify that the identified consensus network was not a product of random chance, we further conducted a permutation test with 5000 iterations by randomly shuffling the diagnostic labels. Based on a consensus threshold of selection in at least 3 out of 5 folds (≥60%), the resulting consensus network achieved a significance level of *p_perm_* < 0.05. Among these, four edges achieved the highest observed support ratio (4/5 folds, support ratio = 0.80). This indicates that the observed level of fold-wise stability is statistically higher than what would be expected by chance, supporting that these complexity-driven prefrontal alterations are robustly associated with the disease stages.

**Figure 7:**
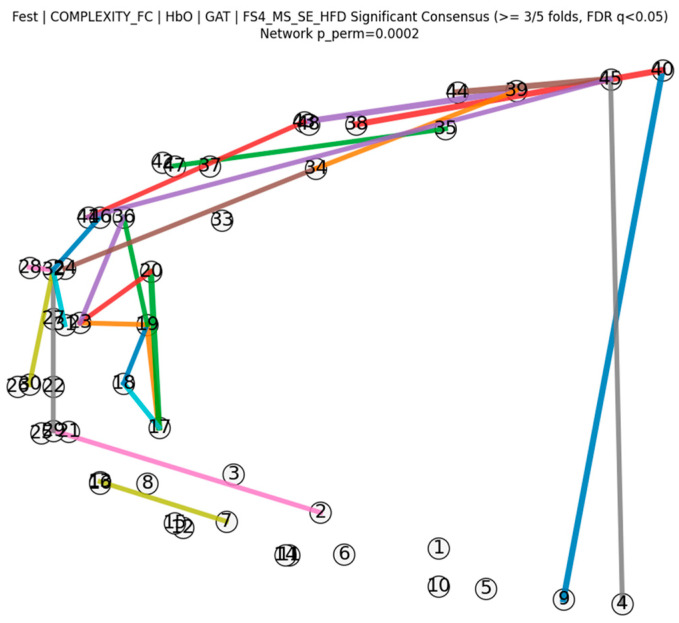
Consensus network across cross-validation folds: Edges shown were selected in ≥3 out of 5 folds and passed edge-level FDR correction (*q* < 0.05); edge thickness denotes fold support. Permutation testing (*p_perm_* < 0.05) was used to evaluate the statistical significance of the consensus network.

Figure 7 presents the resulting statistically significant consensus network, where edge thickness represents fold-support frequency. The consensus edges were spatially concentrated in the left frontopolar, ventrolateral and dorsolateral prefrontal regions and substantially overlapped with the discriminative connections identified in the MCI–HC comparison.

#### 3.3.3. Attention-Difference Network Patterns

**Figure 8:**
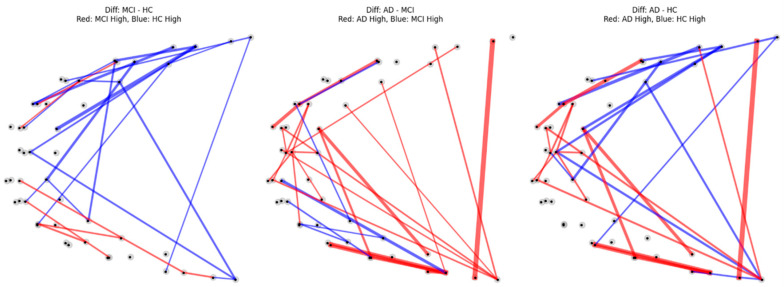
Attention difference networks across diagnostic group comparisons. Edges represent differences in graph attention weights between groups. Red edges indicate connections with higher attention weights in the first-mentioned group (MCI in MCI–HC, AD in AD–MCI and AD–HC comparisons), whereas blue edges indicate connections with higher attention weights in the second-mentioned group. Edge thickness is proportional to the magnitude of the attention-weight difference. Nodes correspond to fNIRS measurement channels.

Attention-difference analysis further characterized how the graph attention network emphasized distinct connections across diagnostic groups. In the MCI vs. HC comparison, widespread HC-dominant connections (blue edges) were observed, consistent with the statistical biomarker analysis. In contrast, AD vs. MCI and AD vs. HC comparisons exhibited sparser and more heterogeneous AD-dominant connections (red edges). The spatial correspondence between attention-difference networks and statistically derived biomarkers was highest in the MCI–HC comparison (Figure 8), supporting the consistency between model-driven attention mechanisms and explicit statistical comparisons.

To verify the functional relevance of the identified biomarkers, we conducted a perturbation analysis by removing the top-10% of edges with the highest attention weights. This resulted in a significant performance drop of ΔMacro-F1 = 0.231. In contrast, removing the same number of edges randomly (N = 1000) yielded a negligible average drop of 0.026. The observed degradation was statistically significant (*p* < 0.01), confirming that the attention mechanism correctly prioritized connections essential for disease stage classification.

## 4. Discussion

In this study, we proposed an interpretable graph neural network framework for Alzheimer’s stage assessment using resting-state prefrontal fNIRS signals, with a particular emphasis on complexity–fluctuation-based network representations. Rather than prioritizing absolute classification accuracy, the present work aimed to identify reproducible, network-level biomarkers that reflect disease-stage-dependent alterations in neurovascular dynamics.

Several key observations emerged from the results. First, overall classification performance remained moderate across models and configurations, reflecting the inherent difficulty of multi-class Alzheimer’s stage decoding from resting-state fNIRS signals. Second, both node feature composition and edge construction strategy substantially influenced model behavior, indicating that graph representation design plays a critical role in extracting disease-relevant information. Third, among the evaluated models, the graph attention network (GAT) provided the most interpretable and internally consistent results, motivating its selection for subsequent biomarker analysis [40,41]. Finally, interpretable graph biomarkers revealed that network-level alterations were most consistently expressed at the MCI stage, whereas AD-related changes appeared more heterogeneous and less statistically separable.

### 4.1. Implications of Graph Representation Design

The present results demonstrate that the choice of graph representation plays a critical role not only in classification performance but also in the structural organization of functional brain networks. By comparing conventional amplitude-based functional connectivity (RAW_FC) with complexity–fluctuation coupling (COMPLEXITY_FC), we showed that improvements in disease-stage discrimination are accompanied by systematic topological reorganization rather than simple reweighting of existing connections.

At the performance level, COMPLEXITY_FC consistently outperformed RAW_FC across multiple feature sets and models, particularly in MCI discrimination. Importantly, this advantage was observed under identical thresholding conditions, indicating that the gain cannot be attributed to differences in network density or edge count alone. Instead, the results suggest that complexity–fluctuation coupling captures inter-regional coordination that is complementary to amplitude-based connectivity.

Topological analysis further revealed that COMPLEXITY_FC networks are characterized by higher local clustering and reduced global integration compared to RAW_FC. Visually, this distinction was reflected in sharper, block-like connectivity patterns in complexity-based adjacency matrices, whereas RAW_FC exhibited more diffusely distributed high connectivity. Quantitatively, COMPLEXITY_FC graphs showed increased clustering coefficient alongside reduced global efficiency and giant component size, indicating a shift toward locally cohesive but globally less integrated network organization.

This topological profile is particularly relevant in the context of mild cognitive impairment. Early neurodegenerative processes are known to disrupt large-scale network integration, while local functional assemblies may transiently strengthen as compensatory mechanisms. Increased local clustering in COMPLEXITY_FC may therefore reflect synchronized fluctuations in nonlinear dynamics within prefrontal subnetworks that remain functionally engaged despite declining long-range communication. Such patterns are consistent with reports of compensatory reorganization in prodromal Alzheimer’s disease and are less readily captured by amplitude-based functional connectivity.

Notably, complexity–fluctuation coupling operates on temporal trajectories of nonlinear signal properties rather than raw hemodynamic amplitudes. As a result, it emphasizes co-modulation of dynamical states across regions, naturally yielding clustered network structures that highlight coordinated changes in complexity. This property provides a mechanistic explanation for why complexity-based graphs are particularly sensitive to early-stage alterations and underscores the importance of representation design in graph-based neuroimaging analyses.

### 4.2. Interpretation of MCI-Dominant Network Alterations

One of the most prominent findings of this study was that the strongest and most reproducible network-level differences were observed between MCI and healthy controls [42,43]. Both statistical edge-importance analysis and attention-difference visualization consistently indicated reduced complexity-based inter-channel coordination in MCI, particularly in left frontopolar, ventrolateral and dorsolateral prefrontal regions [44,45]. This pattern suggests that MCI is characterized by a systematic disruption of coordinated complexity fluctuations rather than a gradual monotonic progression from healthy aging to Alzheimer’s disease [46]. In this context, MCI may represent a critical transitional state in which compensatory mechanisms begin to fail, resulting in detectable but relatively homogeneous network-level alterations. Importantly, this interpretation is supported by the convergence of multiple analytical perspectives: statistical testing, attention-based model introspection, and fold-wise consensus analysis. The spatial overlap between consensus edges and MCI–HC discriminative connections further strengthens the biological plausibility of the identified biomarkers.

### 4.3. Heterogeneity of AD-Related Network Patterns

In contrast to the MCI stage, comparisons involving Alzheimer’s disease (AD) exhibited weaker statistical separation and more heterogeneous network patterns. Although several connections showed higher importance in AD relative to HC or MCI, these effects did not consistently reach statistical significance, and attention-difference networks appeared sparse and spatially variable. This observation should not be interpreted as a failure of the proposed method, but rather as a reflection of the intrinsic heterogeneity of AD [47]. Advanced disease stages are known to involve diverse pathological trajectories, including variable degrees of neuronal loss, vascular dysfunction, and compensatory reorganization [9,14]. As a result, resting-state network signatures may become less uniform across individuals, particularly when measured non-invasively using fNIRS. From a methodological perspective, these findings emphasize that network-level biomarkers derived from resting-state complexity dynamics may be most effective for detecting early or intermediate disease stages, rather than late-stage Alzheimer’s disease.

### 4.4. Stability, Reproducibility, and Model Reliability

A potential concern raised by the fold-wise stability analysis is the relatively low Jaccard similarity observed between sets of important edges across cross-validation folds. However, it is important to emphasize that the stability was consistently non-zero, and a subset of edges was repeatedly selected across folds. In the context of resting-state fNIRS, subject-level graph construction, and limited sample sizes, complete stability across folds is neither expected nor biologically realistic [48,49]. The observed variance in cross-validation performance may be attributed to the intrinsic nature of resting-state dynamics and the pathological complexity of the Alzheimer’s spectrum. Unlike task-based studies with clear event-related triggers, resting-state neurovascular coupling is more susceptible to moment-to-moment fluctuations in brain states and systemic interference. Furthermore, the broad biological heterogeneity in neurovascular compensation, where different patients may exhibit divergent cortical reorganization patterns despite similar cognitive scores, likely contributes to the sensitivity of the GNN models to specific data partitions. Instead, the identification of a small number of consensus edges appearing in a majority of folds represents a meaningful balance between sensitivity and reproducibility. Moreover, the spatial concentration of consensus edges and their overlap with statistically significant MCI–HC biomarkers suggest that the observed stability is not driven by random noise. Rather, the model appears to consistently rely on a limited set of prefrontal connections that carry disease-relevant information.

### 4.5. Relationship Between Model-Driven Attention and Statistical Biomarkers

Another important aspect of this study is the agreement between model-driven attention mechanisms and explicit statistical analyses. In the MCI–HC comparison, attention-difference networks closely mirrored the connections identified through edge-importance statistics, indicating that the GAT model relied on biologically meaningful network features rather than spurious correlations [40,41]. This consistency supports the interpretability of the proposed framework and suggests that attention weights can serve as a complementary tool for biomarker discovery in graph-based neuroimaging analyses [47]. At the same time, the weaker correspondence observed in AD-related comparisons highlights the limits of attention-based interpretation in the presence of highly heterogeneous disease patterns [30]. To address the inherent instability in model training, our framework prioritized the identification of a ‘Consensus Network’ rather than focusing solely on peak classification accuracy. By aggregating important connections that repeatedly appeared across multiple cross-validation folds, we aimed to distill the most reliable prefrontal signatures that are robust to individual variability and potential overfitting.

### 4.6. Limitations

Several limitations of the present study should be acknowledged, along with the methodological choices employed to mitigate their potential impact. First, the sample size, particularly for the Alzheimer’s disease (AD) group, was relatively modest, which may have limited statistical power and contributed to variability in fold-wise biomarker selection. The relatively high variance observed in certain model configurations also points to the sensitivity of hyperparameters to the current sample size. To reduce the influence of this limitation, we employed subject-level graph representations and stratified cross-validation, ensuring that all evaluations were performed on independent subjects and that class imbalance was explicitly accounted for through class-weighted loss functions.

Second, the analysis was restricted to prefrontal fNIRS recordings, and disease-related alterations in other cortical regions were not examined. However, this regional focus was intentionally chosen to minimize inter-subject variability and to target a brain region with well-established relevance to executive dysfunction and early cognitive impairment.

Third, functional connectivity was defined using Pearson correlation of window-wise complexity–fluctuation time series, which captures linear dependencies but does not explicitly model nonlinear or directed interactions. To partially address this limitation, multiple complementary complexity metrics were integrated at the node level, enabling the graph representation to encode nonlinear temporal dynamics even when edge construction remained linear.

Fourth, subject-level graphs were constructed by aggregating window-wise features, potentially smoothing transient dynamics that may carry diagnostic information. To mitigate this effect, sliding windows with high temporal overlap were employed, preserving fine-grained fluctuations in complexity trajectories while maintaining a stable subject-level representation suitable for cross-validation.

Finally, while fold-wise stability of selected graph biomarkers was limited in absolute terms, the analysis consistently identified a subset of consensus edges appearing across multiple folds. Rather than interpreting low absolute stability as a methodological failure, the study explicitly quantified stability using Jaccard similarity and consensus thresholds. This approach provides transparency regarding the reproducibility of the identified network signatures.

### 4.7. Future Directions and Clinical Implications

Future work may address these limitations by incorporating larger and more diverse cohorts, extending the analysis to multi-region fNIRS configurations, and exploring alternative measures of functional coupling, including nonlinear or directed connectivity metrics. In addition, integrating longitudinal data could enable the tracking of individual disease trajectories and the validation of identified biomarkers as predictors of cognitive decline. From a clinical perspective, the present findings suggest that resting-state fNIRS complexity–fluctuation networks may be particularly well suited for early-stage disease assessment. The task-free nature of the proposed framework, combined with its interpretability and modest data requirements, supports its potential integration into longitudinal monitoring systems and closed-loop neurotechnological applications [38].

### 4.8. Interpretation of Results

Most resting-state fNIRS studies in neurodegenerative disorders have focused on static channel-wise features, conventional functional connectivity, or performance-driven classification models, offering limited insight into how disease-related network alterations emerge across cognitive stages. In contrast, the present study introduces an interpretable graph-based framework that models the coordination of time-resolved complexity fluctuations in prefrontal neurovascular signals. By shifting the analytical focus from signal amplitude or static connectivity to network-level synchronization of nonlinear dynamics, this approach captures aspects of resting-state brain function that are not readily accessible through traditional analyses.

A key contribution of this work is its emphasis on reproducible and interpretable network-level biomarker discovery rather than maximal classification accuracy. Using graph attention mechanisms as analytical tools rather than black-box predictors, the proposed framework enables systematic identification and validation of disease-relevant prefrontal network signatures. Importantly, the results indicate that network-level alterations were most consistently expressed at the MCI stage, while AD-related patterns were more heterogeneous. This finding challenges the assumption of monotonic network degradation and supports the view of MCI as a critical transitional state characterized by relatively homogeneous breakdown of coordinated complexity dynamics. Finally, the task-free nature of the proposed framework, together with its modest data requirements and interpretable outputs, highlights its potential relevance for longitudinal monitoring and closed-loop neurotechnological applications. By advancing resting-state fNIRS from descriptive analysis toward mechanistically informed network modeling, this study provides a complementary perspective for understanding and assessing neurodegenerative disease progression.

## 5. Conclusions

This study presented an interpretable graph neural network framework for Alzheimer’s stage assessment using resting-state prefrontal fNIRS signals, with a particular focus on complexity–fluctuation-based network representations. By modeling subject-level graphs that capture coordinated nonlinear dynamics across fNIRS channels, the proposed approach extends resting-state fNIRS analysis beyond conventional channel-wise or static connectivity paradigms. Rather than positioning graph neural networks solely as predictive classifiers, this work formulated disease-stage assessment as a process of automated and stability-aware network-level biomarker identification. Through the integration of fold-wise stability analysis, consensus network identification, and attention-based model introspection, the framework enabled the extraction of interpretable prefrontal network signatures that were consistently implicated in mild cognitive impairment. The results demonstrated that network-level alterations were most reliably expressed at the MCI stage, while patterns associated with Alzheimer’s disease were more heterogeneous. This finding highlights the importance of focusing on early and intermediate disease stages when developing resting-state fNIRS-based diagnostic models and supports the view that MCI represents a critical transition state characterized by systematic disruption of coordinated complexity dynamics.

Overall, this study provides a foundational infrastructure for interpretable and reproducible biomarker discovery from resting-state fNIRS data. By prioritizing network-level consistency and mechanistic interpretability, the proposed framework establishes a necessary methodological basis for future longitudinal studies, multimodal integration, and the development of reliable fNIRS-based diagnostic and monitoring systems for neurodegenerative diseases.

## Figures and Tables

**Table 1:** Demographic information of participants. Data in this table were previously reported in Ref. [38].

Characteristics	AD (*n* = 19)	MCI (*n* = 37)	HC (*n* = 27)	*p*-Value
Sex (male:female)	9:10	13:24	12:15	0.66
Age (mean ± SD)	77.95 ± 6.56	72.23 ± 6.21	66.46 ± 5.67	<0.01 *
Education years (mean ± SD)	9.63 ± 3.60	10.76 ± 3.67	11.14 ± 2.94	0.32
K-MMSE score (mean ± SD)	15.11 ± 1.91	23.84 ± 2.58	28.19 ± 1.44	<0.01 *

* indicates *p*-values with the covariance analysis to compensate for the age difference between groups (SD: standard deviation, AD: Alzheimer’s disease, MCI: mild cognitive impairment, HC: healthy control, K-MMSE: Korean Version of Mini-Mental State Examination).

**Table 2:** Detailed comparison of graph neural network architectures.

Architecture	GCN	GAT	STGNN (GCN + GRU)
Graph Type	Static, homogeneous	Static (homogeneous/heterogeneous)	Dynamic, time-varying
Core Layer Type	Spectral graph convolution	Attention-based message passing	Spatial graph convolution + recurrent temporal unit
Neighborhood Definition	Fixed adjacency matrix	Attention-weighted adjacency	Time-dependent adjacency
Aggregation Operator	Degree-normalized sum	Multi-head attention	Spatial aggregation + GRU temporal update
Normalization	Symmetric degree normalization	Implicit attention normalization (softmax over edges)	Degree normalization (spatial) + gated recurrent normalization
Update Function	Linear transform + ReLU	Linear projection + attention-weighted MLP	GCNConv → GRUCell → GCNConv
Stacking Strategy	Sequential GCN layers	Sequential attention layers	Spatial–temporal alternating layers
Pooling/Readout	Global mean pooling	Global mean pooling	Global mean pooling
Parameter Sharing	Layer-wise	Edge-wise (attention coefficients)	Shared across spatial layers; recurrent temporal parameters
Model Depth	4 layers (hidden 64)	4 layers (heads 4, hidden 64, dropout 0.5)	GCN hidden 64, GRU hidden 64, dropout 0.5
Temporal Kernel	None	None	GRU-based *n* = 3
Spatial Receptive Field	2-hop (*L* = 2)	2-hop (*L* = 2)	2-hop spatial + recurrent temporal integration

**Table 3:** Dataset characteristics of resting-state fNIRS recordings.

Group	Number of Subjects	Recording Length (Samples)	Total Windows	Mean Windows (per Subject)
HC	27	2033	6048	224
MCI	37	2033	8288	224
AD	19	2033	4256	224

**Table 4:** Representative high-sensitivity configurations and model selection rationale.

Model	Edge Type	Hemoglobin	Feature Set	Recall_MCI	Macro-F1	Interpretation
STGNN	COMPLEXITY_FC	Both	FS1	≈1.00	≈0.200	Single-class collapse
GAT	RAW_FC	HbO	FS5	≈1.00	≈0.200	Single-class collapse
GAT	COMPLEXITY_FC	HbO	FS4	0.75–0.85	≈0.846	Balanced sensitivity and overall performance (selected)
GCN	COMPLEXITY_FC	Both	FS4	0.65–0.75	≈0.618	Conservative but stable
STGNN	COMPLEXITY_FC	Both	FS7	0.70–0.80	≈0.735	Balanced sensitivity

## Data Availability

The data are not publicly available due to privacy and ethical restrictions.

## Supplementary Materials

The following supporting information can be downloaded at: https://www.mdpi.com/article/10.3390/brainsci16020239/s1, Table S1: Performance summary across graph representation designs. Figure S1: Detailed performance analysis of the 5-fold cross-validation using the GAT model and complexity–fluctuation coupling (COMPLEXITY_FC) graphs. Figure S2: Detailed performance analysis of the 5-fold cross-validation using the GAT model and complexity–fluctuation coupling (COMPLEXITY_FC) graphs.
