# Supplementary material for: Interpretable Network-Level Biomarker Discovery for Alzheimer’s Stage Assessment Using Resting-State fNIRS Complexity Graphs
Source: Brain Sci. 2026 Feb 19;16(2):239. doi: 10.3390/brainsci16020239 (PMC12938511; doi:10.3390/brainsci16020239)
Supplement: Supplementary file 1 [file brainsci-16-00239-s001.zip › brainsci-4135104-supplementary.pdf]

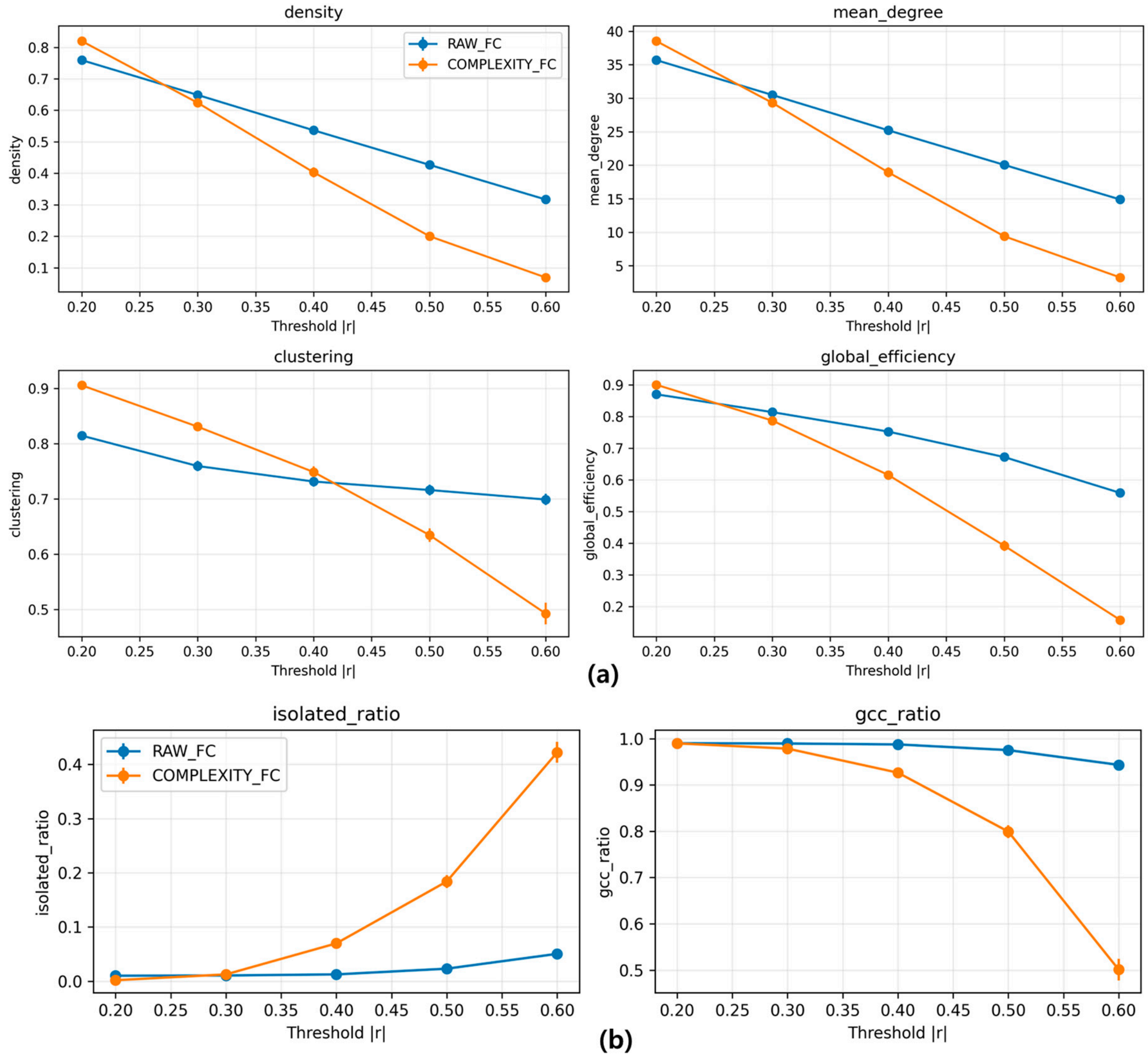

Supplementary Figure S1. Detailed performance analysis of the 5-fold cross-validation using the GAT model and complexity-fluctuation coupling (COMPLEXITY_FC) graphs.

(a) Topological metrics across correlation thresholds: Network density, mean degree, clustering coefficient, and global efficiency were evaluated as a function of the absolute correlation threshold (|r| = 0.2–0.6). Both RAW_FC and COMPLEXITY_FC networks exhibited smooth and monotonic transitions within the range |r| = 0.2–0.4, indicating the absence of abrupt topology shifts. Notably, COMPLEXITY_FC demonstrated higher clustering at lower thresholds, reflecting enhanced local organization.

(b) Fragmentation analysis: The isolated node ratio and giant component ratio (GCC ratio) were computed to assess network integrity. At the selected threshold (|r| = 0.3), the isolated ratio remained near zero and the GCC ratio remained close to 1.0, indicating preserved global connectivity without network fragmentation. Fragmentation became apparent only at higher thresholds (|r| ≥ 0.5), particularly in the COMPLEXITY_FC condition.

Supplementary Table S1. Performance summary across graph representation designs

Classification performance across all combinations of graph neural network models, edge construction strategies, and node feature sets for resting-state fNIRS data (Rest condition). Values are reported as mean ± standard deviation across five-fold cross-validation. Model parameter selection.

Model	Edge type	Feature set	Macro-F1 (mean ± SD)	MCI recall
**GAT**	COMPLEXITY_FC	FS0_MS	0.647 ± 0.023	0.68
**GAT**	COMPLEXITY_FC	FS1_MS_SE	0.627 ± 0.020	0.74
**GAT**	COMPLEXITY_FC	FS2_MS_HFD	0.652 ± 0.026	0.63
**GAT**	COMPLEXITY_FC	FS3_MS_WE	0.607 ± 0.026	0.68
**GAT**	COMPLEXITY_FC	FS4_MS_SE_HFD	0.846 ± 0.060	0.85
**GAT**	COMPLEXITY_FC	FS5_MS_SE_WE	0.650 ± 0.035	0.68
**GAT**	COMPLEXITY_FC	FS6_MS_HFD_WE	0.624 ± 0.022	0.7
**GAT**	COMPLEXITY_FC	FS7_MS_SE_HFD_WE	0.620 ± 0.026	0.64
**GAT**	RAW_FC	FS0_MS	0.537 ± 0.039	0.61
**GAT**	RAW_FC	FS1_MS_SE	0.506 ± 0.037	0.59
**GAT**	RAW_FC	FS2_MS_HFD	0.583 ± 0.024	0.53
**GAT**	RAW_FC	FS3_MS_WE	0.543 ± 0.026	0.59
**GAT**	RAW_FC	FS4_MS_SE_HFD	0.710 ± 0.040	0.7
**GAT**	RAW_FC	FS5_MS_SE_WE	0.200 ± 0.020	1
**GAT**	RAW_FC	FS6_MS_HFD_WE	0.530 ± 0.022	0.6
**GAT**	RAW_FC	FS7_MS_SE_HFD_WE	0.503 ± 0.038	0.54
**GCN**	COMPLEXITY_FC	FS0_MS	0.682 ± 0.039	0.78
**GCN**	COMPLEXITY_FC	FS1_MS_SE	0.629 ± 0.021	0.6
**GCN**	COMPLEXITY_FC	FS2_MS_HFD	0.625 ± 0.026	0.73
**GCN**	COMPLEXITY_FC	FS3_MS_WE	0.663 ± 0.035	0.58
**GCN**	COMPLEXITY_FC	FS4_MS_SE_HFD	0.618 ± 0.110	0.7
**GCN**	COMPLEXITY_FC	FS5_MS_SE_WE	0.648 ± 0.027	0.69
**GCN**	COMPLEXITY_FC	FS6_MS_HFD_WE	0.645 ± 0.038	0.71
**GCN**	COMPLEXITY_FC	FS7_MS_SE_HFD_WE	0.618 ± 0.035	0.71
**GCN**	RAW_FC	FS0_MS	0.555 ± 0.024	0.65
**GCN**	RAW_FC	FS1_MS_SE	0.560 ± 0.038	0.51
**GCN**	RAW_FC	FS2_MS_HFD	0.539 ± 0.025	0.62

**GCN**	RAW_FC	FS3_MS_WE	0.514 ± 0.036	0.51
**GCN**	RAW_FC	FS4_MS_SE_HFD	0.520 ± 0.050	0.55
**GCN**	RAW_FC	FS5_MS_SE_WE	0.536 ± 0.022	0.63
**GCN**	RAW_FC	FS6_MS_HFD_WE	0.531 ± 0.027	0.61
**GCN**	RAW_FC	FS7_MS_SE_HFD_WE	0.512 ± 0.034	0.61
**STGNN**	COMPLEXITY_FC	FS0_MS	0.613 ± 0.021	0.62
**STGNN**	COMPLEXITY_FC	FS1_MS_SE	0.200 ± 0.020	1
**STGNN**	COMPLEXITY_FC	FS2_MS_HFD	0.589 ± 0.039	0.67
**STGNN**	COMPLEXITY_FC	FS3_MS_WE	0.632 ± 0.038	0.72
**STGNN**	COMPLEXITY_FC	FS4_MS_SE_HFD	0.642 ± 0.025	0.64
**STGNN**	COMPLEXITY_FC	FS5_MS_SE_WE	0.683 ± 0.028	0.57
**STGNN**	COMPLEXITY_FC	FS6_MS_HFD_WE	0.594 ± 0.030	0.76
**STGNN**	COMPLEXITY_FC	FS7_MS_SE_HFD_WE	0.735 ± 0.090	0.75
**STGNN**	RAW_FC	FS0_MS	0.552 ± 0.029	0.5
**STGNN**	RAW_FC	FS1_MS_SE	0.190 ± 0.025	1
**STGNN**	RAW_FC	FS2_MS_HFD	0.523 ± 0.022	0.54
**STGNN**	RAW_FC	FS3_MS_WE	0.563 ± 0.037	0.62
**STGNN**	RAW_FC	FS4_MS_SE_HFD	0.581 ± 0.038	0.55
**STGNN**	RAW_FC	FS5_MS_SE_WE	0.582 ± 0.037	0.5
**STGNN**	RAW_FC	FS6_MS_HFD_WE	0.512 ± 0.027	0.64
**STGNN**	RAW_FC	FS7_MS_SE_HFD_WE	0.640 ± 0.040	0.6

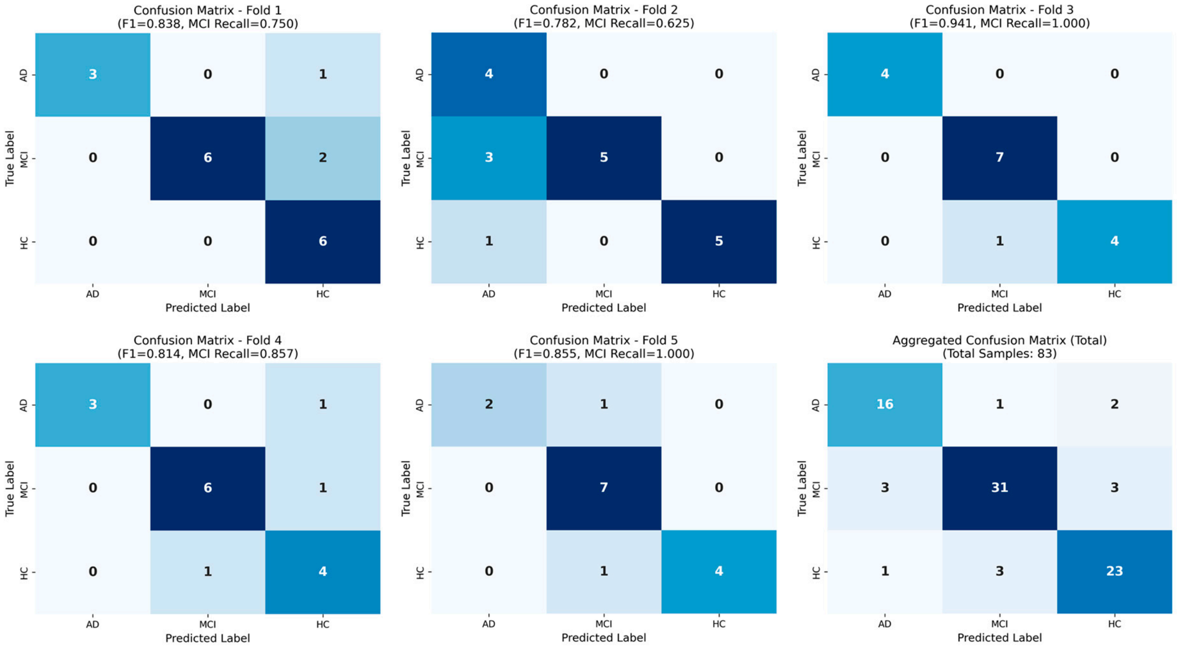

Supplementary Figure S2. Detailed performance analysis of the 5-fold cross-validation using the GAT model and complexity-fluctuation coupling (COMPLEXITY_FC) graphs.
